# A Multicenter Evaluation of Overall Susceptibility and Antimicrobial Resistance Among Streptococcus pneumoniae Isolates

**DOI:** 10.7759/cureus.41984

**Published:** 2023-07-17

**Authors:** Namrata Kulkarni, Abhisek Routray, Santosh Taur

**Affiliations:** 1 Department of Medical Affairs, Pfizer Limited, Mumbai, IND

**Keywords:** vaccine, resistance, susceptibility, antibiotics, pneumococcal disease, streptococcus pneumoniae

## Abstract

Purpose: *S. pneumoniae* ranks as the fourth-most lethal pathogen globally in terms of fatalities associated with or attributable to resistance. In this study, the Antimicrobial Testing Leadership and Surveillance (ATLAS) analysis from India aims to study the overall antimicrobial susceptibility (AMS) among pneumococcal isolates collected between 2018 and 2021.

Methods: Non-duplicate clinically significant *S. pneumoniae* isolates were collected between 2018 and 2021. In vitro activity of antibiotics was assessed against *S. pneumoniae.* Susceptibility was confirmed at an International Health Management Associates (IHMA) laboratory using supplied broth microdilution panels (Omron Microscan Systems, Inc., Omron Corp., Kyoto, Japan), according to the Clinical and Laboratory Standards Institute (CLSI) guidelines for all antibiotics.

Results: Of the total 86 non−duplicate isolates of *Streptococcus pneumoniae* collected from the tertiary care centers, the proportion of isolates increased from 8.14% (n=7) in 2018 to 43.02% (n=37) in 2020. Most isolates (n = 18; 48.65%) were collected from the age group of 31-60 years in the year 2020. Erythromycin revealed a decrease in susceptibility from the year 2018 (71.43%) to 2020 (16.22%). A decreased susceptibility of 90% was recorded for levofloxacin in the year 2021. Meropenem revealed a decrease in susceptibility from the year 2018 (85.71%) to 2020 (35.14%). Penicillin susceptibility decreased from 37.5% in 2019 to 27.03% in the year 2020. Clindamycin indicated a 100% susceptibility in the year 2018 which then decreased to 71.88% in 2019 and 56.76% in 2020. Linezolid and vancomycin were found to have uniform susceptibility of 100% throughout the years from 2018 to 2021.

Conclusion: An increase in resistance to penicillin and macrolides among *S. pneumoniae *isolates was observed in the Indian population. Addressing the elevating rates of *S. pneumoniae *resistance may require pneumococcal conjugate vaccines (PCVs) with expanded serotype coverage and targeted antimicrobial stewardship efforts.

## Introduction

Pneumococcal disease refers to any infection caused by lancet-shaped, Gram-positive, facultative anaerobic bacteria called *Streptococcus pneumoniae*, or pneumococcus [1, 2]. It has more than 100 known serotypes and is a major cause of morbidity and mortality in developing countries [1, 3]. Invasive pneumococcal disease (IPD) is defined as the isolation of bacteria from a normally sterile site (cerebrospinal fluid, blood, and pericardial fluid) and includes infections such as bacteremia, septicemia, osteomyelitis, septic arthritis, pneumonia associated with bacteremia, and meningitis. Non-invasive pneumococcal diseases occur outside the major organs or the blood and include less serious infections such as bronchitis, otitis media, community-acquired pneumonia (CAP), and sinusitis [4-6]. According to estimates from the World Health Organization (WHO), *S. pneumoniae* kills more than 300,000 children under the age of five worldwide every year [7]. IPD incidence was found to be 7 per 100,000 people in England and Wales for all age categories, rising to 21 per 100,000 for people over 65 years old [8]. IPD case mortality rates can range from 15%-20% in adults to 30%-40% in the elderly [8]. India was estimated to have the highest burden of pneumococcal deaths in 2015 [9].

Beta-lactam antibiotics such as penicillin and third-generation cephalosporins are regarded as the mainstay of treatment for *S. pneumoniae* infections. Fluoroquinolones (e.g., levofloxacin), lincosamides (e.g., clindamycin), macrolides (e.g., erythromycin), and vancomycin can be used to treat beta-lactam non-susceptible pneumococcal isolates and for individuals who are unable to tolerate beta-lactams [10]. Penicillin has been the drug of choice for CAP since its introduction in 1943 [11]. In a landmark study published in 1964, Austrian and Gold demonstrated that there was a decrease in mortality resulting from bacteremic pneumococcal pneumonia from 80% in untreated patients to 17% in patients with penicillin therapy [11].

In 1967, the first clinically significant isolate of resistant *S. pneumoniae* was isolated in Australia [11]. Drug-resistant *S. pneumoniae *has become a subject of global concern, with high levels of resistance reported in some European and Southeast Asian countries [12]. Pneumococcus was listed as one of the nine bacteria of international concern in a WHO report on antibiotic resistance that was released in 2014 [13]. A number of variables have been identified as the root cause leading to the development of antimicrobial resistance (AMR), including the widespread availability of antibiotics as over-the-counter (OTC) medications in some countries, irrational prescriptions by medical practitioners, and non-compliance by patients [10]. The study by Van Boeckel et al. reported that between 2000 and 2010, the global consumption of antibiotics increased by more than 30%, from around 50 billion to 70 billion standard units. India consumed the most antibiotics in 2010 with 13 billion standard units, followed by China with 10 billion and the United States with 7 billion standard units (a standard unit is the number of doses sold; the IMS Health database identifies a dose as a pill, capsule, or ampoule) [13]. *S. pneumoniae* is an extremely challenging pathogen to manage because of the increased utilization of antibiotics, the spreading of several resistant clones, the ability of pneumococcus to undergo serotype replacement and capsular switching, and the capability of horizontal transmission of antibiotic resistance genes [13]. According to the Centers for Disease Control and Prevention (CDC), *S. pneumoniae* isolates that are resistant to one or more clinically significant antibiotics are estimated to be responsible for about 30% of IPD cases. The CDC has classified drug-resistant *S. pneumoniae* isolates as a serious threat after they caused an estimated 900,000 infections and 3,600 deaths per year in all age groups in 2014. *S. pneumoniae* ranks as the fourth most lethal pathogen globally in terms of fatalities associated with or attributable to resistance [14].

Understanding the significance of antibiotic therapy practices in the emergence of resistance and revising the empiric therapy guidelines for pneumococcal disease at a national and regional level can be accomplished by examining AMR trends among pneumococcal isolates [10]. It is particularly important to document potential differences in AMR patterns for *S. pneumoniae*, given the geographic diversity of India [12]. Thus, ATLAS analysis from India aims to study the prevalence and temporal trends of antimicrobial susceptibility (AMS) among pneumococcal isolates collected from multiple centers between 2018 and 2021.

To address AMR, disease prevention is an important measure. Vaccines could mitigate or avoid life-threatening diseases, as well as decrease medical expenses and post-infection complications. Additionally, it could reduce the need for antibiotics (both first- and second-line treatments), which could prevent the development of AMR [15].

## Materials and methods

Bacterial strains

Non-duplicate isolates of *Streptococcus pneumoniae* (n = 86) collected from multiple centers representing different geographies of India between 2018 and 2021 were included. Demographic information recorded for each isolate included specimen source, and patient age, sex, and location in the hospital. To be included in the study, isolates had to be considered the likely causative pathogen of infection. Isolates were collected from various clinical samples including blood, sputum, bronchoalveolar lavage, endotracheal aspirate, bronchus, intestine, urine, thoracentesis fluid, gastric abscess, gall bladder, and pancreas. Species identification was performed at each participating centers using routine local methodologies.

The central repository for all isolates was International Health Management Associates (IHMA, Schaumburg, IL, US [1]), who were responsible for organism transport and collection, confirmation of isolate identification and the management of a database including all isolate data.

Antimicrobial susceptibility testing

All isolates were identified locally and shipped to the central laboratory (International Health Management Associates, Inc., Schaumburg, IL, US) for susceptibility testing. The Clinical and Laboratory Standards Institute (CLSI) recommended broth microdilution method (BMD) was used to assess the susceptibility of *Streptococcus pneumoniae* to various antibiotics (Table 1) [16]. MIC data were used only if daily QC results were within acceptable ranges as published by the CLSI.

## Results

Total number of isolates

**Figure 1:**
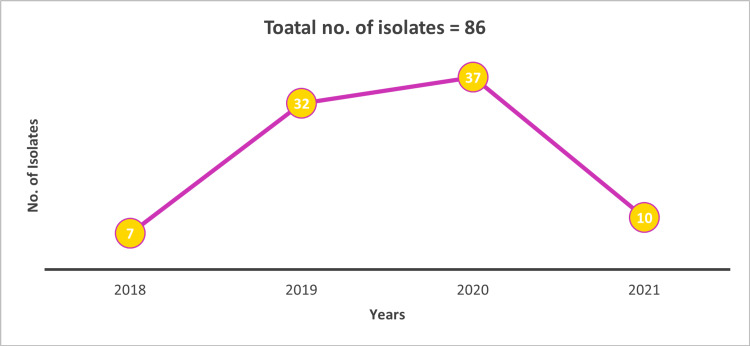
Total No. of Isolates

**Figure 2:**
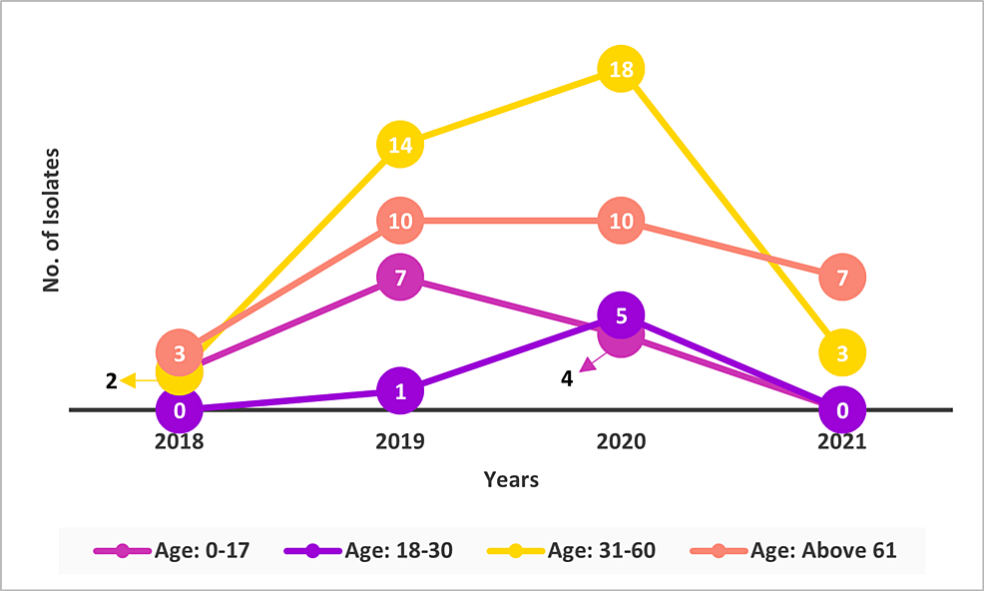
Age-wise – No. of Isolates

A total of 86 non-duplicate isolates of *Streptococcus pneumoniae* were collected from multiple centers representing different geographies of India between the years 2018 and 2021. The proportion of *Streptococcus pneumoniae* increased from 8.14% (n=7) in 2018 to 43.02% (n=37) in 2020. Approximately, 11.63% (n = 10) of *Streptococcus pneumoniae* isolates were collected in the year 2021 (Figure 1). A maximum number of isolates (n = 18; 48.65%) were collected from the age group of 31-60 years in the year 2020 (Figure 2).

Antibacterial susceptibility

Table 1 shows the antibacterial susceptibility of the antibiotics included in this study. Ceftaroline was found to have complete susceptibility of 100% in the years 2018, 2020, and 2021 and a susceptibility of 96.88% in the year 2019. Ceftriaxone showed an increase in susceptibility from the year 2018 (57.14%) to 2021 (90%). Clindamycin indicated a 100% susceptibility in the year 2018 which then decreased to 71.88% in 2019 and 56.76% in 2020; in the year 2021, a slight increase in susceptibility (60%) was observed.

**Table 1:** Antibacterial Susceptibility

Antibacterial	2018 (N=7)			2019 (N=32)			2020 (N=37)			2021 (N=10)		
	Susceptible [N(%)]	Intermediate [N(%)]	Resistant [N(%)]	Susceptible [N(%)]	Intermediate [N(%)]	Resistant [N(%)]	Susceptible [N(%)]	Intermediate [N(%)]	Resistant [N(%)]	Susceptible [N(%)]	Intermediate [N(%)]	Resistant [N(%)]
Ceftaroline	7 (100%)	0 (0%)	0 (0%)	31 (96.88%)	1 (3.13%)	0 (0%)	37 (100%)	0 (0%)	0 (0%)	10 (100%)	0 (0%)	0 (0%)
Ceftriaxone	4 (57.14%)	2 (28.57%)	1 (14.29%)	28 (87.5%)	3(9.38%)	1 (3.13%)	32 (86.49%)	4 (10.81%)	1 (2.70%)	9 (90%)	1 (10%)	0 (0%)
Clindamycin	7 (100%)	0 (0%)	0 (0%)	23 (71.88%)	1 (3.13%)	8 (25%)	21 (56.76%)	1 (2.70%)	15 (40.54%)	6 (60%)	0 (0%)	4 (40%)
Erythromycin	5 (71.43%)	0 (0%)	2 (28.57%)	11 (34.38%)	0 (0%)	21 (65.63%)	6 (16.22%)	1 (2.70%)	30 (81.08%)	2 (20%)	0 (0%)	8 (80%)
Levofloxacin	6 (85.71%	1 (14.29%)	0 (0%)	30 (93.75%)	0 (0%)	2 (6.25%)	37 (100%)	0 (0%)	0 (0%)	9 (90%)	0 (0%)	1 (10%)
Linezolid	7 (100%)	0 (0%)	0 (0%)	32 (100%)	0 (0%)	0 (0%)	37 (100%)	0 (0%)	0 (0%)	10 (100%)	0 (0%)	0 (0%)
Meropenem	6 (85.71%	0 (0%)	1 (14.29%)	26 (81.25%)	4 (12.5%)	2 (6.25%)	13 (35.14%)	18 (48.65%)	6 (16.22%)	6 (60%)	3 (30%)	1 (10%)
Penicillin	0 (0%)	6 (85.71%)	1 (14.29%)	12 (37.5%)	14 (43.75%)	6 (18.75%)	10 (27.03%)	7 (18.92%)	20 (54.05%)	3 (30%)	5 (50%)	2 (20%)
Vancomycin	7 (100%)	0 (0%)	0 (0%)	32 (100%)	0 (0%)	0 (0%)	37 (100%)	0 (0%)	0 (0%)	10 (100%)	0 (0%)	0 (0%)

Erythromycin revealed a decrease in susceptibility from the year 2018 (71.43%) to 2020 (16.22%); in the year 2021, a slight increase in susceptibility (20%) was observed. Levofloxacin susceptibility increased from the year 2018 (85.71%) to reach 100% susceptibility in the year 2020. However, a decreased susceptibility of 90% was recorded for levofloxacin in the year 2021. Linezolid was found to have uniform susceptibility of 100% throughout the years from 2018 to 2021.

**Figure 3:**
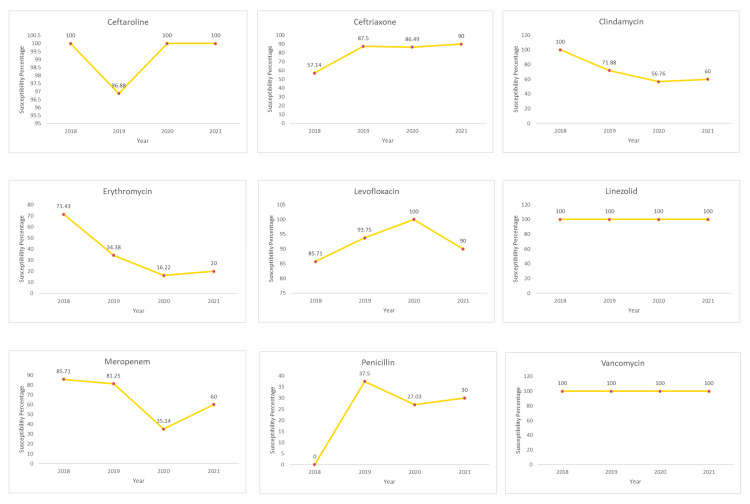
Antibacterial Susceptibility of the Antibiotics

Meropenem revealed a decrease in susceptibility from the year 2018 (85.71%) to 2020 (35.14%); in the year 2021, a slight increase in susceptibility (60%) was observed. Penicillin showed 0% susceptibility to *Streptococcus pneumoniae* in the year 2018. In the year 2019, penicillin showed an increased susceptibility of 37.5% which then decreased to 27.03% in the year 2020. Penicillin recorded a susceptibility of 30% in the year 2021. Vancomycin was found to have uniform susceptibility of 100% throughout the years from 2018 to 2021 (Figure 3).

Sources of isolates

**Figure 4:**
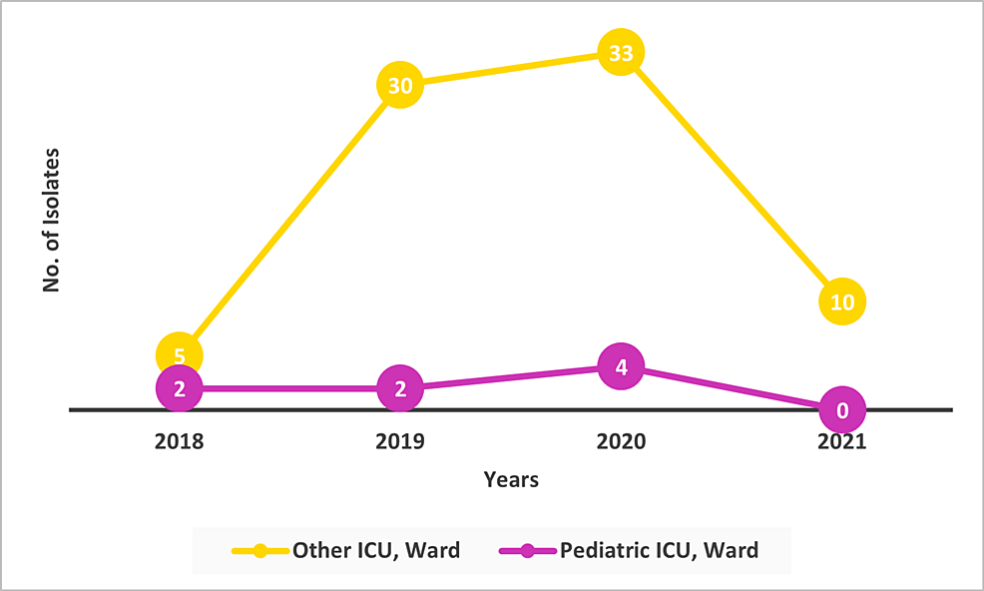
Ward-wise – No. of Isolates

**Figure 5:**
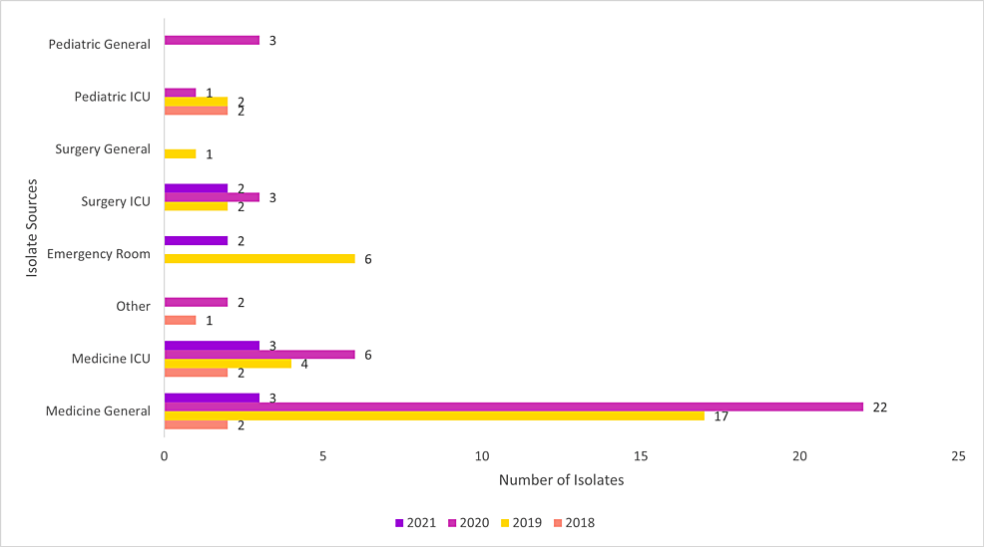
Isolate Sources

A maximum number of 33 isolates (42.31%) were collected from sources such as Medicine ICU, Medicine General, Surgery ICU, and Other in the year 2020 (Figures 4-5).

## Discussion

Despite considerable decline in disease incidence with the introduction of PCVs, *S. pneumoniae* continues to be a significant contributor to pediatric and adult infections [14, 17]. In adults, pneumonia is the primary cause of pneumococcal infections. According to epidemiological studies from the European region that were conducted among adults, the incidence rate of CAP ranged from 1.6 to 11.6/1000 people. According to the WHO 2008 study, Asia had the highest prevalence of pneumonia, with the Indian subcontinent bearing the greatest burden of the disease. Additionally, findings from an Indian prospective study showed that CAP was the second-most common cause of infectious disease-related death in the region [5]. The risk of acquiring pneumonia caused by *S. pneumoniae* and pneumococcal invasive disease is also increased by influenza virus infection. According to a study conducted during the 2009 H1N1 pandemic, pneumococcal hospitalizations were found to be increased at the time of highest pandemic influenza activity. The largest relative rise in pneumococcal hospitalizations was seen in children and adolescents aged 5 to 19 years, who have low baseline levels of pneumococcal disease (ratio 1.6; 95% CI 1.4-1.7), while the largest absolute increase was seen in people aged 40 to 64 years [14, 17]. ATLAS analysis from India aimed to study the prevalence and temporal trends of susceptibility among pneumococcal isolates.

In this multicenter surveillance study conducted for a span of four years from the year 2018 to 2021, a total of 86 *Streptococcus pneumoniae* isolates were collected. In this study, a maximum number of isolates (n = 18; 48.65%) were collected from the age group of 31-60 years in the year 2020 which indicates that *Streptococcus pneumoniae *infections are not limited to the pediatric population. Older individuals experience complicated immune system changes with aging (immunosenescence), making them more susceptible to pneumococcal and other infectious illnesses. Additionally, the elderly adult population typically has one or more chronic comorbidities that exacerbate the condition, as opposed to young adults. In this population, the primary focus should be on pneumococcal disease vaccination because it is an essential preventive measure [5]. A lesser number of isolates were observed in 2021 when compared to the years 2019 and 2020 because people continued to socially isolate because of ongoing SARS-CoV-2 infections and the emergence of more transmissible variants. Other COVID-19 control strategies, such as wearing a mask and maintaining social distance, could have also played a role to limit the spread of pathogens in the year 2021, particularly in healthcare facilities.

Overall susceptibility to *Streptococcus pneumoniae* isolates was reported for the following antimicrobial classes in this study: cephalosporins, lincosamides, macrolides, fluoroquinolones, oxazolidinones, carbapenems, penicillins, and glycopeptides. In our study, an increasing resistance was observed for lincomycin, macrolide, carbapenem, and beta-lactam class of antibiotics with the lowest susceptibilities recorded as 56.76%, 16.22%, 35.14%, and 27.03%, respectively in the year 2020. Our findings are consistent with Aliberti et al. study which reported that the most common AMR found in subjects with drug-resistant *Streptococcus pneumoniae* community-acquired pneumonia (DRSP-CAP) was macrolide resistance (0.6%), followed by penicillin resistance (0.5%) [18]. In the study of Mohanty et al., *S. pneumoniae* isolates from US children with IPD and non-invasive PD infections exhibit high levels of resistance to macrolides and penicillin [14].

WHO has described the term "antimicrobial stewardship (AMS)" as coordinated efforts to encourage the optimal use of antibiotics, including the choice of antibiotics, the decision to use them, dosage, route, and duration of administration. The ten interventions coined by WHO as a part of AMS include (1) clinician education; (2) patient and public education; (3) institution-specific guidelines for the management of common infections; (4) cumulative antibiograms; (5) prior authorization of restricted antimicrobials; (6) de-labeling of spurious antibiotic allergies; (7) prospective audit and feedback; (8) self-directed antibiotic reassessments (antibiotics timeouts); (9) dose optimization; (10) duration optimization [19].

All antibiotic-resistant isolates cannot be treated effectively with vaccines but *S. pneumoniae* infections constitute one of the few antibiotic-resistant threats that can be effectively mitigated by vaccines [14]. The primary vaccine-preventable cause of death in children under five years old globally and in India is pneumococcal disease. The inclusion of pneumococcal vaccinations in the national immunization program is justified since they currently appear to be the only public health tool capable of significantly reducing the burden of pneumococcal diseases in India [20]. By minimizing the prevalence of *S. pneumoniae* serotypes that are more likely to be resistant to antibiotics and by reducing respiratory illnesses that lead to antibiotic use, pneumococcal vaccinations can be used to prevent AMR [14]. Clinical studies have demonstrated that PCV is 80% effective in preventing invasive pneumococcal illness caused by the bacterial serotypes contained in the vaccine formulation. Children who receive vaccinations have a 27% lower risk of pneumonia diagnosis and an 11% lower risk of pneumonia-related death [21]. In a population-based investigation that lasted for two years, it was found that CAP patients who had previously received the PPSV23 vaccination had a 40% lower death or intensive care unit admission rate than nonvaccinated patients [22]. In a retrospective analysis of older COPD patients, those who received vaccinations had lower hospitalization rates, lower mortality rates, and lower direct medical care costs. The use of vaccines was linked to a 43% decrease in pneumonia hospitalizations [23]. Additionally, PCV13 may be able to slow the growth of antibiotic resistance. The vaccination achieves this effect by reducing the spread of pneumococcal serotypes (19A) that are resistant to antibiotics and by preventing the disease from occurring in the first place, thereby eliminating the need for antibiotics [24]. In a lab-based investigation, a decrease in the prevalence of infections among older adults who are resistant to antibiotics was also noted. The incidence rate of pneumococcal illnesses driven by penicillin-resistant strains dropped to 8.4 from 16.4 cases per 100,000 people. In addition, the prevalence of resistant pneumococcal disease due to vaccine serotypes reduced by 87% [25]. In India, PCV is administered in children in three doses (two as primary doses and one as a booster dose) at six weeks, 14 weeks, and nine months. However, India has not yet fully implemented adult vaccination programs and these vaccines are underutilized since there is no clear mandate requiring all adults to receive vaccinations [26, 27]. Although the vaccines are advocated for and recommended globally for the elderly population at risk, their use in the region is suboptimal and heavily dependent on clinicians' knowledge of guidelines [5].

Our study has limitations, the primary being that our investigations focused primarily on isolates that tested positive for culture, and the existence of symptomatic pneumococcal disease was not established. Another limitation of this study is the relatively small sample size which did not allow us to investigate the potential differences in serotypes, strains, and antibiotic resistance among *S. pneumoniae*. Due to an increased possibility of testing more severely ill patients, selection bias is a potential issue that may increase estimates of resistance. We did not assess the patients' pneumococcal immunization status which would have been beneficial additions to a subsequent analysis. The evaluation of the pneumococcal strains present in India was prevented by the paucity of serotyping and genotyping results. Other potential limitations include the lack of information on associated infections. There were no statistics on nosocomial versus community-acquired infections in our analysis. Future research addressing the problem of pneumococcal epidemiology in the nation should take all these constraints into consideration while developing their study designs.

## Conclusions

In conclusion, our data document increasing resistance to penicillin and macrolides in *S. pneumoniae *isolates among the Indian population. These data may help inform initial treatment decisions for outpatients and inpatients with suspected pneumococcal infections. The rates of resistance to macrolides have increased in *S. pneumoniae* isolates over the past four years, despite overall disease reductions attributable to vaccines. Addressing the elevating rates of *S. pneumoniae* resistance may require PCVs with expanded serotype coverage and targeted antimicrobial stewardship efforts. The absence of established standards for the use of pneumococcal vaccine in the region, however, may be the cause of the country's inadequate pneumococcal vaccination rates. There is a need for campaigns to raise awareness among healthcare providers' understanding of vaccine recommendations.
